# Suffering doubly: Effect of cyberbullying on interpersonal deviance and dual mediating effects of emotional exhaustion and anger

**DOI:** 10.3389/fpsyg.2022.941235

**Published:** 2022-11-29

**Authors:** Nausheen Syed, Abu Bakar Abdul Hamid, Xin Su, Misbah Hayat Bhatti

**Affiliations:** ^1^PUTRA Business School, Seri Kembangan, Selangor, Malaysia; ^2^Department of Management Sciences, Government College Women University, Faisalabad, Pakistan; ^3^School of Economics and Management, Beijing University of Posts and Telecommunications, Beijing, China

**Keywords:** workplace bullying, employee harassment, emotional exhaustion, anger, conservation of resource theory, affective event theory

## Abstract

Research on employee harassment, in the form of workplace bullying, has increased over the past decade. However, there is little research on the prevalence and impact of cyberbullying, a type of cyber-related violence in the workplace. Thus, it would be interesting to examine the impact of cyberbullying on interpersonal deviance through the serial mediating effect of emotional exhaustion and anger. Drawing from the conservation of the resource theory and the affective event theory, this proposed study clarifies the mediating effects of emotional exhaustion and anger. The time lag approach was used to collect the data from the sample of 385 employees in the telecommunication sector of Pakistan. By employing SPSS and PLS, bootstrapping was performed to conduct the mediation analysis. Findings indicated that workplace cyberbullying increased interpersonal deviance by enhancing emotional exhaustion and anger. The current research contributes to the literature by considering the behavioral outcomes of workplace cyberbullying with the practical implications for human resource practitioners.

## Introduction

High-tech outbreak has affected the workplace in both positive and negative ways. An upsurge of numerous harmful activities emerges due to extensive access to information & communication technology (ICT). Incidence of cyberbullying rises exponentially (Zhang S. et al., 2021). Cyberbullying is stated as a hostile and deliberate act of perpetrators using ICT to send threatening messages frequently to the other person to whom it is herculean to entrench (Vranjes et al., 2017). Personal communications of the victim may be shared publicly, which may disturb his/her personal and working life. Cyberbullying is an explicit global delinquent act that affects the normal working of organizations all over the globe, such as the USA, UK, Canada, Australia, and New Zealand (Snyman and Loh, 2015). Pakistan as a developing country has the largest technology user, which shows that its people are also affected by cyberbullying activities. The extent of cyberbullying is not restricted to one’s personal and social dwelling, but its remnants have lurked into the places of work in various sectors (Vranjes et al., 2018a; Choi and Park, 2019).

Researchers found the drastic impact of cyberbullying on the victim’s cerebral predilections and social consequences (Fahy et al., 2016; Koay, 2018; Jiang et al., 2020). Fahy et al. (2016) initiated positive interaction between cyberbullying and depression among adolescents. Cyberbullying is more annoying than face-to-face bullying.

Cyberbullying victims are inclined to evolve from the offensive online behavior and move toward counterproductive work behavior, which was further investigated by prior research (Kowalski et al., 2017; Vranjes et al., 2018a). Scholars have dedicated their attention toward cyberbullying, but limited studies attempted to explore its impact in the workplace setting and interpersonal outcomes (Coyne et al., 2017; Park and Choi, 2019). The existing body of cyberbullying has focused on the influence of the previous experience of being cyberbullied, and the target of the current review is to explore the strike back by workplace cyberbullying by pandering in interpersonal deviance, which may comprise behaviors such as the use of verbal abuse, sharing offensive comments, and publicly humiliating colleagues (Michalak et al., 2018; Anwar et al., 2020).

This is elaborated by the affective event theory (Weiss and Cropanzano, 1996), which proposes that workplace occurrences induce volatile harmful sentiments among operatives. Different work stressors experienced by employees on a regular interval impact their emotions, and workplace cyberbullying is one such stressing factor. Workplace cyberbullying creates frustration among employees that indulge them in interpersonal deviance (Zhang S. et al., 2021; Zhang Z. et al., 2021). Coyne et al. (2017) demonstrated that faculty members in the UK frequently received one to three unethical emails and tended to display strong job dissatisfaction. Similarly, Li et al. (2021) also reconnoitered daily oscillation in interpersonal deviance. Victims of workplace cyberbullying are less prudent to elucidate the emotional issues in the short run (Schilpzand et al., 2016). Earlier research failed to scrutinize within-person discrepancies in workplace cyberbullying and preys who were unable to probe the dynamic process, makeing them aggressive after experiencing workplace cyberbullying (Rosen et al., 2016). Hence, investigation of the potential effect of workplace cyberbullying on employee interpersonal deviance can extend our understanding of its distractive influences on organizations, especially in the telecommunication sector of Pakistan.

The effect of cyberbullying in the workplace on interpersonal deviance can be erected through emotional exhaustion, which can occur when an individual has a lack of emotional resources that are a basic requisite for handling interpersonal stressors. It is one of the dimensions of burnout and includes the depletion of emotional resources, conviction, interest, and loss of concern (Maslach and Jackson, 1981). Conservation of resource theory (Hobfoll, 2011) is overwhelmingly important as it gives an overarching context through which to try to make things clear on how workplace cyberbullying may yield reserve loss and detrimental consequences for dupes. In the terms of harmful outcomes, the center of attention will be the victim’s feeling of emotional exhaustion as an instantaneous significance of victim behavior. Emotionally exhausted employees have reduced emotional resources which makes it grim for them to execute the interpersonal and job demands. For example, they intentionally put efforts to slow down the work, smudge the organization’s reputation, and damage organizational property (Jahanzeb and Fatima, 2017). In the light of COR theory, the present study considers the impact of workplace cyberbullying as a type of workplace stressor on interpersonal deviance. Victims of cyberbullying are more likely to involve in interpersonal deviance and a lack of resources leads to emotional exhaustion.

Workplace cyberbullying is a premeditated aggressive behavior that occurs repetitively over time through technologies between an offender and a target who has inadequate power (Wang et al., 2019). Bullying may prompt a sense of anger among employees, a high vivification emotion that reflects belligerent reactions. Anger pushes employees to engage in harmful acts such as interpersonal and organizational deviance (Vranjes et al., 2017). Present inquiry inducement on affective event theory (Weiss and Cropanzano, 1996) states that affective events might form an employee’s behavior by evoking their effective reactions. Affective event theory (AET) postulates that sentiments are acquiescent to momentous vacillations as a function of an individual’s day-to-day experience. When the core values of employees are under threat, they experience anger. Workplace cyberbullying violates the fundamental moral values of employees that fuel anger toward the perpetrator and induce them toward interpersonal deviance (Li et al., 2021). Therefore, we envisage that anger toward the perpetrator mediates the impact of workplace cyberbullying on interpersonal deviance.

By doing so, our study makes important contributions in three folds. First, we have deepened our understanding of the consequences of workplace cyberbullying and its effect on interpersonal deviance at an episodic level by incorporating perceptive from the AET. Second, our inquisition of novel strain instructive mechanism emotional exhaustion allows us to scrutinize interpersonal deviance as an adverse outcome of workplace cyberbullying from a viewpoint of COR. Third, emotions have preponderantly been investigated as a repercussion of workplace cyberbullying and a gauge of interpersonal deviance. Therefore, in response to a request for further research on the role of individual emotions in the context of important stressors in the workplace, we include anger as an intermediary for this previously ignored relationship (Park and Lewis, 2019).

## Literature review and hypothesis development

### Workplace cyberbullying

The power of ICT is available to every workstation and every organizational level in today’s technology-driven organizations. When there is no proper check and regulation on ICT, it can be a sort of disaster that can drop the organizational productivity and profitability. As the workplace changes, so do patterns of harassment. Workplace harassment is accepted as an urgent problem faced by both employers and employees (Oksanen et al., 2020; Barlett et al., 2021).

Cyberbullying at work is an idiosyncratic experience for victims, reflecting their longstanding observations of aggressive behavior as demonstrated by ICT use in the workplace. From this type of bullying, it is easy for the victims to feel helpless and defenseless (Farley et al., 2016; Vranjes et al., 2018a). Cyberbullying is becoming a global social issue with an expansion of social networking services. However, cyberbullying studies are limited to adolescents and children; workplace cyberbullying has become petite distention (Lee, 2014; Farley et al., 2016).

According to D’cruz and Noronha (2013), boundarylessness and pervasiveness of negative behavior is an important leitmotif in the cyberbullying experience in the workplace. This kind of behavior invades an individual’s personal life and makes them feel pursued. Shifting all the work activities to the technology during COVID-19, 24/7 availability of victims, and a constant connection cannot escape this negative behavior. Coyne et al. (2017) conducted a study on British university employees and concluded that 8 out of 10 employees experience workplace cyberbullying in the last 6 months. About 14% to 20% became victims of these acts on at least a weekly basis. Victims of cyberbullying report higher depression and lower organizational commitment. Due to technology gratification, this issue has become more powerful and ubiquitous in the workplace. D’Souza et al. (2022) has stated that cyberbullying was positively related to the employee’s intention to leave, job dissatisfaction, and anxiety.

### Interpersonal deviance

Deviant workplace behaviors are serious concerns for organizations, threatening its internal operations and external competitive advantage. These kinds of deviant work activities have a negative impact on perpetrators, undermining their performance evaluations and career development (Zhang et al., 2018; Valle et al., 2019). Even knowing the implications of negative effects for themselves, why do employees decide to engage in interpersonal deviance?

Interpersonal deviance includes intentional deviant behavior targeted toward individuals (such as malicious talk, ferocity, and theft from coworkers), which disrupts the normal working of the organization and threatens the wellbeing of victims. Examples of such behaviors include vocal exploitation, offensiveness, and racial harassment (Bennett and Robinson, 2000). Nourishing auspicious interpersonal relationships and obstructing aggressive behavior requires harnessing one’s limited reserves for control (Koopman et al., 2020). Interpersonal deviance occurs when employees do not have the cognitive resources to do so. These employees may feel anxious about their work environment and fear for their career growth, so it may seem tempting to express their frustrations (Azeem et al., 2021).

Interpersonal deviance diminishes employees’ ruminations concerning their structure functioning, and as a result, such forms of responses create a less dangerous feel about their precarious state of affairs. Previous studies elaborated that interpersonal deviance is negatively related to organizational citizenship behavior and wellbeing (Markova, 2018; Eissa et al., 2019).

### Workplace cyberbullying and interpersonal deviance

Workplace cyberbullying is defined as the use of ICT for sending threatening messages to the victims and making things problematic, especially for the accomplishment of tasks and publicly forwarding the confidential communications of victims (Vranjes et al., 2018a). Previous investigations revealed that workplace cyberbullying indulges employees in counterproductive work behavior. Consequently, cyber victimization employees become deviant (Anwar et al., 2020; Akram et al., 2022). Cyberbullying targeting specific individuals can unswervingly induce interpersonal relationships among employees (Giumetti et al., 2012). Hence, the focus of the present study is to examine whether victims of cyberbullying in the workplace retaliate by engaging in interpersonal deviance.

Interpersonal deviance is a clear distinction intended to harm individuals versus organizational deviance to the detriment of the organization (Bennett and Robinson, 2000). Cyberbullying at work is positively related to international deviance because workplace cyberbullying depletes victims’ cognitive resources, and these recourses engage vicitims in negative provoking trends (Park et al., 2018). Sufficient evidence has been provided by Rosen et al. (2016) that, when employees practice workplace cyberbullying, they must disburse, attentional sources to manage, the emotional burdens and frustration by indulging in interpersonal deviance. Based on the above discussion, the following hypothesis is proposed:

**H1:** *Workplace cyberbullying is associated with interpersonal deviance*

### Mediating effect of emotional exhaustion

Emotional exhaustion as a basis syndrome of burnout is defined as the depletion of emotional resources among individuals and reserves of low energy (Maslach et al., 2001). An insufferable state of emotional exhaustion could lead victims to a state of tiredness and depression (Xu et al., 2015). Frustrated individuals tend to be involved in counterproductive work behavior to release this frustration. The current literature related to emotional exhaustion proposes that it can affect the behavior of a variety of employees such as deviant behavior (Jiang et al., 2020) and negligence behavior (Aliza et al., 2021).

According to the conservation of resource theory (Hobfoll, 2001), employees who experience pressure will first regulate whether not they have ample resources to alter their strain. If they are unable to cope and their reserves are repetitively depleted without redemption, they provoke a harmful emotional and psychological reaction. Among these, emotional exhaustion is the most common undesirable response to the disagreeable. A study by Zhang Z. et al. (2021) indicated that workplace cyberbullying is a form of interpersonal stressor that leads to emotional exhaustion, and as a result, employees begin to involve in destructive activities, such as interpersonal deviance, to release themselves from the annoying situation. Stressor-emotion model developed by Spector and Fox (2005) demonstrated the importance of emotions as an antiphon to workplace nuisance. Emotions lead to interpersonal deviance that can transpire impetuously or at a subsequent time. Empirical evidence provided support that emotional exhaustion can activate interpersonal deviance. In particular, workplace cyberbullying might arouse interpersonal deviance as a surviving strategy that permits employees to conceal their frustration that their hiring organization confesses colleague’s exploitation within its levels.

Victims of workplace cyberbullying experience emotional exhaustion because they have reduced cognitive, emotional, and psychological resources, which makes it strenuous for them to admonish work and interpersonal demands, for example, using abusive language, gossip, and intentionally sharing employees’ personal information publicly. The avoidance coping approach is being used by employees to detach themselves from occupational duties and engross themselves in interpersonal deviance (Jiang et al., 2021). Emotionally exhausted employees do not have self-regulatory resources to end deviant behavior but are also more likely to gridlock their enduring reserves and evade capitalizing supply in behavioral adjustments. Consequently, inquiry has demonstrated that emotional exhaustion is positivity narrated to interpersonal behaviors (Kong et al., 2018). Therefore, in the view of COR theory, emotionally exhausted employees are more likely to spend limited resources in response to interpersonal deviance in response to workplace cyberbullying. Accordingly, we hypothesize the following:

**H2:** *Workplace cyberbullying and interpersonal deviance are mediated by emotional exhaustion.*

### Mediating effect of anger

Employees at the workplace spend most of their time interacting with supervisors and co-workers who are experiencing different emotions. Among them, anger is an emotion with varying intensities, from mild arousal to rage (Spielberger et al., 1985). Organizational observers particularly focus on anger expression that pushes the employees toward deviant behavior. Geddes and Callister (2007) are particularly interested in observing expressions of anger that push employees toward deviant behavior such as intentionally sending detestable messages to a coworker, verbal abuse, and fighting with others.

Research in psychology demonstrates that angry employees directly abuse their partners because they blame the other persons for unwelcome circumstances. In such cases, employees may involve civic conduct and contain anomalous behaviors targeted by individuals who develop interpersonal deviations, e.g., theft from colleagues and violent gossip (Schwarzmüller et al., 2017). According to the AET theory (Weiss and Cropanzano, 1996), work stress experienced by employees affected them emotionally and psychologically, thereby stimulating employee performance. Thus, exploring the association between cyberbullying and interpersonal deviance is like testing a black box, as it cannot shed light on changes in the psychology and emotions of people in the form of anger.

Studies by Miner et al. (2005) demonstrated that emotion-focused behaviors must scrutinize employee emotional responses. Undesirable emotions tempted negative behavior. Anger caused by workplace cyberbullying can lead to revengeful conduct. Deviance is defined as a volunteer conduct that disrupts substantial organizational values and threatens the welfare of the organization (Heerdink et al., 2013).

According to AET (Weiss and Cropanzano, 1996), once employees encounter emotion-stimulating incidents in an organization, their sentiments are aroused and are consequently associated with anger, which could be a felt sentiment when a hostile incident happens. In line with AET (Weiss and Cropanzano, 1996), victims of workplace cyberbullying may perceive organizational management as incapable of protecting them from bullies. The bully will practice indicators of anger and fuel the fight for the regulator. Especially, employees may display temporary anger by involving in interpersonal deviance in the reaction to workplace cyberbullying (Jahanzeb et al., 2020). For example, if the victim has fear that retaliation against the organization might jeopardize his/her promotion opportunities, then the victim may shift his/her anger toward the co-workers (Achyldurdyyeva et al., 2021). Considering the positive relationship between anger and workplace cyberbullying, we propose the following hypothesis:

**H3:** *Anger mediates the relationship between workplace cyberbullying and interpersonal deviance.*

## Research methodology

### Sample and procedure

This study scrutinizes the relationship between workplace cyberbullying and interpersonal deviance through serial mediating effects of emotional exhaustion and anger. Pakistan’s telecommunication sector has made immense progress in the terms of brands and a large consumer base, so we have collected data from this sector. The selection of the Pakistani telecom sector is based on numerous logics. First, nowadays, in technology-driven organizations, the power of ICT is available on each desktop. Instead of ICT being spelled as portability and productivity, it is responsible for all ingredients of disaster. Methods of harassment are changing as the workplace is changing. During the COVID-19 pandemic, employees in the telecommunication sector faced serious workplace cyberbullying. Second, a large base of customers and high competition among telecom brands reduce the employee’s tolerance level and make them emotionally exhausted and angry, which lead them to interpersonal deviance.

Employees of three leading companies, i.e., Mobilink, Telenor, and Ufone, have been approached for data collection. Sales and Marketing, Information technology, and customer operators’ departments have been selected. Due to the extensive branch networks of these companies, employee data collection can be obtained easyily when visiting different outlets. Formal approvals were mandatory from the human resource managers before data collection. A purposive sampling approach was applied for the selection of respondents. Before the circulation of questionnaires, researchers visited employees separately to clarify the purpose and procedure for administrating the survey. We randomly selected employees to participate voluntarily in the survey and guaranteed that all personal information would be kept personal and solely utilized for speculative investigation purposes.

We used a multi-wave design where cause and effect were temporally separated by at least 3 weeks (Podsakoff et al., 2012). Workplace cyberbullying was measured at time 1, emotional exhaustion and anger were tapped at time 2, and interpersonal deviance was measured at time 3. From a total of 500 questionnaires, 385 usable questionnaires were finalized after discarding the incomplete responses. Survey participants belonged to diverse backgrounds and had different levels of management. The majority of the respondents were men (75%) and the remaining respondents were women. Most of them were married (60%) and 40% were single. In terms of functional area, the respondents belonged to the sale and marketing (40%), information technology (45%), and customer operators departments (15%). All of the participants had a university education with 79.8% holding a master’s degree and the rest had a bachelor’s degree (20.2%). The respondent had an average age of 30.2 years (SD = 8.59), an average tenure of 3.45 years (SD = 4.42), and the average total work experience of 5.35 years (SD = 6.31).

### Measures

Development of questionnaires was made in English. Questionnaires did not interpret Urdu as English is the predominant means of certified communication mostly used in the workplace and also teaching language for higher education programs in Pakistan, given that most of the respondents in our sample had university-level education. Past studies have indicated that in Pakistan the use of English questionnaires is no longer problematic (Naseer et al., 2016; Sarwar et al., 2019). Respondents respond on a five-point Likert scale, ranging from 1 = strongly disagree to 5 = strongly agree (Table 1).

**TABLE 1:** Scale description.

Scale	No of items	Source
Workplace cyberbullying (T1)	10	Vranjes et al., 2018b
Interpersonal deviance (T3)	8	Bennett and Robinson, 2000
Emotional exhaustion (T2)	9	Maslach et al., 2001
Anger (T2)	5	Buss and Perry, 1992

### Workplace cyberbullying (T1)

Vranjes et al. (2018b) 10-item scale was used to measure Cyberbullying Acts at the workplace to recognize the undesirable behaviors through information communication technology, such as “gossips are being spread about you using ICT.”

### Interpersonal deviance (T3)

The 8-item scale of Bennett and Robinson (2000) was used to measure interpersonal deviance. Items include “acting rudely toward someone at work.”

### Emotional exhaustion (T2)

Emotional exhaustion is measured on a 9-item scale by Maslach et al. (2001). The sample item included “I feel emotionally drained from my work.”

### Anger (T2)

Employee anger was measured on a 5-item scale developed by Buss and Perry (1992). A measure used to assess the severity of an employee’s anger at the workplace, including emotional components of aggressive emotion. Sample items included “if I get frustrated, I let you know my frustration” and “Sometimes I bend for no good reason and burn quickly, but I get over it quickly.”

### Control and socio-demographic variables

The purpose of this study was to scrutinize if employees who experienced cyberbullying showed interpersonal deviations. Peng et al. (2016) reported that men are more involved in interpersonal deviance than women. Furthermore, according to the theory of socio-emotional selectivity (Carstensen, 1992), older workers are less likely to engross in interpersonal deviance than younger workers. Therefore, in this study, we specified age as a control variable. We also controlled the positive and negative effects.

### Statistical analysis

The present study ran the preliminary analysis including descriptive analysis by using Statistical Packages for the Social Sciences (SPSS; version 26). We conducted Smart PLS (SEM3.3.2, Ringle and Sarstedt, 2016). PLS-SEM has been applied preferably for various reasons. First, it is efficaciously considered for exploratory and prediction-based research. Second, it allows for the analysis of complex models with multiple indicators, constructs, and relationships (Ringle et al., 2018). Third, emotional exhaustion and anger are treated as second-order constructs; hence, PLS-SEM is considered a better choice for dealing with models that have high-order constructs. By following the guideline from PLS-SEM literature (Henseler et al., 2009; Siyal et al., 2019), we adopted the two-step approach to analyze the results. In the first step, we analyzed the measurement model to evaluate the inter-item reliability, composite reliability, and convergent validity. In the second step, we scrutinized the structural model to test the hypothesis and predictive capability assessment (Henseler et al., 2009).

## Results

### Assessment of measurement model

Through the method described by Dijkstra and Henseler (2015), inter-item reliability was assessed. Composite reliability and Cronbach alpha for all variables demonstrated good reliability with a threshold value exceeding 0.70 (Hair et al., 2017). Formerly, factor loadings of all variables have been estimated by maintaining the threshold of 0.70 (Hair et al., 2017). Convergent validity was evaluated through the analysis of average variance extracted (AVE), and all the variables met good convergent validity exceeding the threshold value of 0.50 (Bagozzi et al., 1991). Table 2 demonstrates the results of the measurement model.

**TABLE 2:** Constructs reliability and validity.

Latent variable	Factor loading	Cronbach’s alpha	Dijkstra Henseler’spA	(AVE)	Mean scores
WCB	0.73–0.80	0.89	0.847	0.623	4.32–2.56
ID	0.76–0.82	0.90	0.832	0.737	5.22–3.89
EE	0.85–0.90	0.85	0.867	0.772	5.78–3.45
AG	0.83–0.95	0.82	0.878	0.765	5.62–3.69

WCB, workplace cyberbullying; ID, interpersonal deviance; EE, emotional exhaustion; AG = anger.

Fornell–lacker criterion and Heterotrait-Monotrait (HTMT) interference criterion were used to assess the discriminant validity. Table 3 demonstrates that the square root of AVE must be greater than the inter-correlation values, which means that there is discriminant validity among constructs (Fornell and Larcker, 1981). The recommended value for HTMT was up to 0.85 when the constructs have a higher conceptual distinction (Henseler et al., 2015). If the HTMT is greater than the value of 0.85, it shows the existence of discriminant validity issues. Heterotrait-Monotrait values are below the most conservative threshold of 0.85, confirming the discriminant validity between each pair of constructs (Voorhees et al., 2016). Table 4 demonstrated that the HTMT values of all the variables are above the threshold.

**TABLE 3:** Correlation matrix and discriminant validity.

No	Variables	1	2	3	4	5	6	7	8	9	10
1	Age										
2	Gender	0.136**									
3	Tenure	0.289**	0.297**								
4	Educational level	0.378**	0.135**	0.389**							
5	PA	0.383*	0.378*	0.236**	0.386**						
6	NA	0.267*	0.390**	0.233*	0.486**	0.224*					
7	WCB	0.192**	0.345**	0.395**	0.446*	0.221**	0.395**	**0.775**			
8	ID	0.294*	0.256**	0.237*	0.484*	0.245**	0.346**	0.573**	**0.756**		
9	EE	0.206**	0.255*	0.367**	0.319**	0.342*	0.376*	0.256**	0.465**	**0.885**	
10	AG	0.145**	0.279*	0.286*	0.198*	0.282*	0.489*	0.387**	0.534**	0.499**	**0.849**

The *p*-value is significant at ^**^0.01 and *0.05.; Diagonal values are square roots of AVE that should be higher than the inter-correlation for the estimation of discriminate validity. Bold values indicate the discriminate validity.

**TABLE 4:** Discriminant validity.

	Heterotrait–Monotrait ratio (HTMT)			

Constructs	WCB	ID	EE	AG
WCB	–			
ID	0.698	–		
EE	0.463	0.539	–	
AG	0.554	0.597	0.736	–

### Assessment of the structural model

**FIGURE 1:**
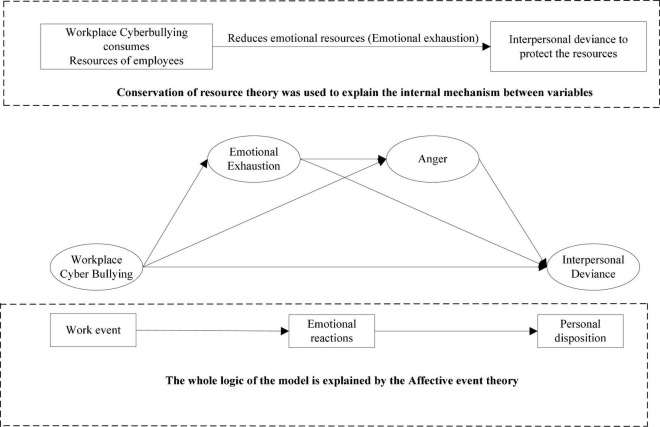
Emotion reaction model of workplace cyber bullying.

A total of 5,000 bootstrapping resamples were used to regulate the significance of path co-efficient for a satisfactory outer model assessment result. Table 5 and 1ures 1A,B demonstrate the structural path coefficient along with t-values and confidence interval. The total effect of (c) workplace cyberbullying on interpersonal deviance is positively significant (β = 0.385, *p* < 0.05; Figure 1A), hence supporting H1.

**TABLE 5:** Effects on endogenous variables.

Effects on endogenous variables	Direct effect	*t*-Value	Percentile bootstrap 95% CI
Emotional exhaustion (*R*^2^ = 0.67/*Q*^2^ = 0.187)			
Workplace cyberbullying(a1)	0.367***	10.234	[0.127;0.156] Sig.
Anger (*R*^2^ = 0.55/*Q*^2^ = 0.178)			
Workplace cyberbullying (a2)	0.389***	4.457	[0.135;0.187] Sig.
Emotional exhaustion (a3)	0.438***	3.756	[0.156;0.178] Sig.
Interpersonal deviance (*R*^2^ = 0.85/*Q*^2^ = 0.274)			
Workplace cyberbullying (c’)	0.027ns	0.397	[0.003; 0.128] N.Sig.
Emotional exhaustion (b1)	0.354***	5.335	[0.148; 0.165] Sig.
Anger (b2)	0.348***	5.567	[0.146;0.173] Sig.

Sig. denotes a significant direct effect at 0.05; ns: not significant. Bootstrapping based on n = 5,000 subsamples.

The effects of workplace cyberbullying on emotional exhaustion (a1) = (β = 0.367, *p* < 0.05) and workplace cyberbullying on anger (a2) = (β = 0.389, *p* < 0.05) are significant. Similarly, the relationship of emotional exhaustion and anger (a3) = (β = 0.438, *p* < 0.05), emotional exhaustion and interpersonal deviance (b1) = (β = 0.354, *p* < 0.05), and anger and interpersonal deviance (b2) = (β = 0.348, *p* < 0.05) are significant. In the mediation model, the direct effect (c’) between workplace cyberbullying and interpersonal deviance (β = 0.027, *p* < 0.05) is not significant.

### Bootstrap mediation analysis

**FIGURE 2:**
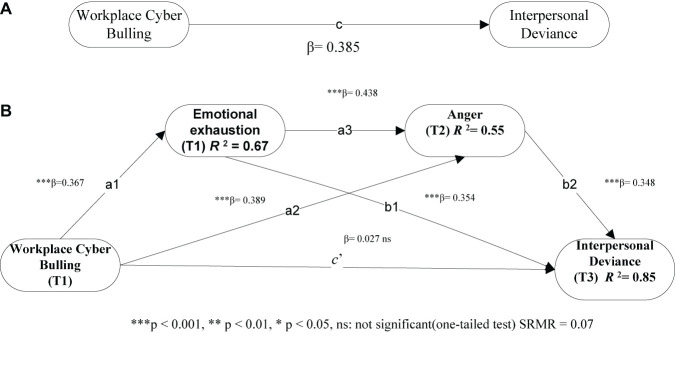
Structural model: Total effect model **(A)** and Three-path mediated model **(B)**.

Nitzl et al.’s (2016) approach was used for the assessment of our mediation hypothesis (H2, H3, and H4). The estimation of all indirect effects of hypotheses is shown in Table 6. The bootstrapping method was applied to assess indirect effects using bias-corrected confidence. As seen in Table 6 and (Figure 2), the direct effect of workplace cyberbullying on interpersonal deviance becomes insignificant (β = 0.027, *p* < 0.05) and its impact becomes insignificant after the inclusion of mediators. Furthermore, all indirect effects of workplace cyberbullying on interpersonal deviance were significant.

**TABLE 6:** Bootstrap mediating results.

	Coefficient	t-value
Total effect of workplace cyberbullying on interpersonal deviance(c)	0.385	0.246
Direct effect of workplace cyberbullying on interpersonal deviance (c’)	0.027(ns)	0.234

**Indirect effects of ethical leadership on knowledge sharing**	**Estimate points**	**Bootstrap 95% CI**

H2: WCB—EE—ID (a1b1)	0.45	[0.176; 0.223]
H3: WCB—AG—ID (a2b2)	0.30	[0.132; 0.267]
H4: WCB—EE—AG—ID(a1a3b2)	0.49	[0.156; 0.176]
Total indirect effect	0.124	[0.149; 0.146]

**p < 0.05, ns, not significant (one-tailed test). WCB, workplace cyberbullying; ID, interpersonal deviance; EE, emotional exhaustion; AG, anger. This symbol indicates the level of significance *0.01.

As seen in Table 6, emotional exhaustion mediated the relationship between workplace cyberbullying and interpersonal deviance. Bias-corrected bootstrap at 95% confidence interval (a1b1 = 0.45, *p* < 0.05; LLCI = 0.176, ULCI = 0.223) confirmed that indirect effect is significant, hence supporting H2. Likewise, anger is a preferential mediator in the relationship between workplace cyberbullying and interpersonal deviance. A significant indirect effect confirmed (a2b2 = 0.29, *p* < 0.05; LLCI = 0.132, ULCI = 0.267) that H3 is supported. When two mediators are introduced, the indirect effects of workplace cyberbullying and interpersonal deviance are significant (a1a3b2 = 0.49, *p* < 0.05, LLCI = 0.156, ULCI = 0.176), hence lending support to H4. In addition, bias-corrected bootstrap at 95% confidence interval confirms that the total indirect effect between workplace cyberbullying and interpersonal deviance is significant (a1b1 + a2b2 + a1a3b2 = 1.24, *p* < 0.05, Table 6). Workplace cyberbullying and interpersonal deviance relationship are fully mediated by emotional exhaustion and anger (large mediation effect, *f*^2^ = 0.55).

*R*^2^, SRMR (standardized “root” means square residual), GOF, and Q^2^ (predictive relevance of endogenous variable) are calculated for the completion of the structural model. *R*^2^ values for emotional exhaustion, anger, and interpersonal deviance were 0.67, 0.55, and 0.85, respectively, which are above the threshold value of 0.10. It shows excellent explanatory power (Hair et al., 2017). Following the guidelines of Henseler et al. (2016), our research model achieves a value of 0.06 which was an appropriate fit, taking the cutoff value of 0.08 into account, and the GOF value is estimated to validate the PLS model through a formula GOF = √AVE × R^2^ = 0.67, which achieves a good model fit. Q^2^ values for emotional exhaustion, anger, and interpersonal deviance were 0.168, 0.187, and 0.254, and all of these values are greater than 0, which indicates strong predictive power for our model.

## Discussion

The determination of this study is to scrutinize whether workplace cyberbullying in an organization could be one of the reasons that victims engross in workplace deviant behavior. According to AET and COR theories, with the help of ICT, employees who were victimized become emotionally exhausted and angry and hence engage in interpersonal deviance.

First, the outcome of this study confirms the positive relationship between workplace cyberbullying and interpersonal deviance (β = 0.278, *p* < 0.01), indicating that the vast media audience, anonymity of perpetrators, and indiscrimination access to the victims at all times made it possible to do workplace cyberbullying at all places. Workplace cyberbullying creates stress that could affect the individual’s mental and physical health in the longer term. Consequently, victims may involve in interpersonal deviance. The outcome of the present study is in line with the prior study by Zhang S. et al. (2021) and Zhang Z. et al. (2021).

Second, the relationship between workplace cyberbullying and interpersonal deviance is mediated by emotional exhaustion (β = 0.45, *p* < 0.01). The findings highlight the trigger effect of emotional exhaustion as a response to workplace cyberbullying. Application of conservation of resource theory helps to define the employee’s menacing situation such as workplace cyberbullying, which may end up evolving harmful emotions (emotional exhaustion) that can lead to demonstrating interpersonal deviance. Consequently, workplace cyberbullied employees may dissipate their emotional and cognitive resources which could sooner or later create emotional exhaustion (Peng et al., 2016). The result of the current study is consistent with the previous study by Anwar et al. (2020).

Third, the findings confirm the mediating effect of anger on the relationship between workplace bullying and interpersonal deviance (β = 0.30, *p* < 0.01). Victims of workplace cyberbullying develop anger that pushes them to involve in interpersonal behavior. Angry employees permutate their anger toward the defenseless targets through intimate gestures in their social environment to retort against perpetrators. We found support from the affective event theory, which states that the negative experiences caused by harmful emotions influence a victim’s behavior. Hence, anger caused by workplace cyberbullying can lead to interpersonal deviance. The outcome of the study is in line with the prior study by Michalak et al. (2018) who demonstrated that the intermediate effect of anger that arises through workplace cyberbullying may end up involving interpersonal deviance.

Finally, the outcome of the study confirms the serial mediating effects of emotional exhaustion and anger in the relationship between workplace cyberbullying and interpersonal deviance (β = 0.49, *p* < 0.01). Emotionally exhausted employees lack emotional and psychological resources, employ an avoidance-focused coping strategy, and ultimately engage in interpersonal deviance. Emotional exhaustion then leads to anger, a form of severe emotion where both mediators simultaneously affect the association between workplace cyberbullying and interpersonal deviance.

### Theoretical implications

The present study has numerous theoretical implications. The current study enriched the evolving literature around workplace cyberbullying and provided a serial mediation model that suggested emotional exhaustion and anger uplifting interpersonal deviance. By incorporating COR theory and AET theory, we propose that workplace cyberbullying demonstrates an enduring form of trauma given in nature (amplification and obscurity). Earlier studies found that tenacious stressors can make an adverse impact on the organization and employee’s physical and mental health and brings hostile job outcomes.

First, workplace cyberbullying urges employees toward interpersonal deviance and augments our consideration of the consequences of workplace cyberbullying at the episodic level. Existing literature reveals that, in the online environment, intrusiveness is related to workplace cyberbullying unlike face-to-face bullying (Vranjes et al., 2018a). Prior investigation has demonstrated that iniquitousness and boundarylessness of negative behavior were the significant themes in the victim’s considerate of workplace cyberbullying (Vranjes et al., 2018a). By considering it, the present study has instigated to discover the outcome of cyberbullying. Intra-person outcomes have been probed by most of the studies (Snyman and Loh, 2015; Coyne et al., 2017). This recent literature has neglected the possible consequences of interpersonal deviance as they do not portray an acceptable illustration of the harmful consequences of workplace cyberbullying. Hence, a vigorous view is essential to excavate our perceptions hooked on how workplace cyberbullying induces harmful behavior outcomes.

Second, scrutinizing the mediating role of emotional exhaustion and demonstrating the fundamental reasoning apparatus through which workplace cyberbullying induces interpersonal deviance. Our research conceptualized on conservation of resource theory which addresses the mediating impact of emotional exhaustion in the relationship between social context and behavioral outcomes. Our research findings contribute to the COR theory by signifying that workplace cyberbullying yields a disagreeable setting that declines employees’ inadequate reserves. Therefore, negative social cues lead to unethical behavior. Self-control resources are limited when employees face cyberbullying which push them to engage in interpersonal deviance.

Third, by employing the affective event theory, the mediating effect of anger on the relationship between workplace cyberbullying and interpersonal deviance is examined. Prior similar studies specify that the relationship of workplace cyberbullying with job attitudes such as job satisfaction and turnover are mediated by the victim’s emotive experience (Coyne et al., 2017, Park and Choi, 2019). Our research study advances the anger literature by incorporating the AET theory, which envisages that harmful sentimental incidents trigger undesirable emotional responses, which in sequence lead to hostile behavior. Consequently, tormented employees practice their anger to initiate their aberrant behavior as apparatus to deal with disgracing executive conduct.

### Managerial implications

The telecommunication sector of Pakistan widely uses ICT, which gives rise to workplace cyberbullying. Our verdicts have numerous practical influences that can guide human resource practitioners in the telecommunication sector to make them able to diminish the undesirable influence of workplace cyberbullying.

First, due to the growing usage of social media within organizations in Pakistan, practitioners are suggested to ponder how online communications could distress employee development. Considering it seriously, human resource managers are responsible to contemplate how to legalize employees’ online interaction by using equivalence of the digital footmark to testify the possible consequences of online exploits. Moreover, civilized user parameters for work-related social media should be circulated in the workplace. Supervisors must try to eradicate workplace cyberbullying proactively by promoting on-the-job training. That training should make every employee aware of how to eradicate the negative consequences of workplace cyberbullying. An organization should also provide interpersonal training skills to any victims of workplace cyberbullying.

Second, to avoid the emotional exhaustion among employees which arises due to workplace bullying, human resource managers should take necessary steps, such as launching an impartial method for grievances against perpetrators. Furthermore, a frequently used amendment technique of bullying is divulging in someone. Therefore, we propose that the human resource manager should promote mental sessions to stimulate employees to speak up about his/her emotions and behaviors of perpetrators.

Third, the mediating role of anger in the relationship between workplace cyberbullying and interpersonal deviance makes human resource managers clear about the reaction of victims toward the negative situation of the organization. Managers must understand that employees’ anger is not always inherently hostile to the specialist. As an alternative, employees’ anger could indicate the existence of deteriorated workplace ailments that need attention. Executives must acquire employees’ anger as a conceivable form of discord in response to a belligerent workplace situation. They can retort with a problem-solving slant in an emotionally enduring way. It must be indistinct that the anger-apparated detrimental actions are logistically intolerable. Therefore, managers encourage the employees to participate in anger management programs so that they can get benefits of leveraging their harmful emotions through prolific work events, such as innovative work behavior and promotive voice, and engender novel ideas for how to detect and eliminate workplace cyberbullying.

### Limitations and future research directions

This study has certain constraints. First, the perpetrators of bullying could be supervisors and co-workers (Fox and Stallworth, 2005). Bullies have diverse characteristics that may affect the victims’ emotional and behavioral responses. Supplementary conductions could inspect how the position of perpetrators affects interpersonal deviance as well as compare with other springs of workplace adversity such as abusive leadership.

Our research model was assessed through the cross-sectional data. Therefore, causation is deduced from the hypothetical lens of conservation of resource theory and AET. In the experiential setting to validate such interconnection, future studies should utilize other practices such as the diary method. This method not only permits investigators to track the responses of respondents in an ordinary background but also alleviates the observation issue by using the time span between when the incident happens and when it is recalled.

The present research study investigates the respondents from the telecommunication sector of Pakistan and as such may not be a representative of all industries’ incidents of cyberbullying. Future studies must include diverse industries such as banking, hotels, and universities, both public and private, to investigate cyberbullying incidents to make it more imperative.

Since this survey was conducted in Pakistan, it is not clear whether the results of this study can be regarded as representative of employee behavior in other countries with diverse culture and working conditions. Future scholars studying in this area may choose to study similar dynamics for themselves or for cultural contexts.

## Conclusion

The current study promotes the existing research by demonstrating how workplace cyberbullying predicts interpersonal deviance through the serial mediating effect of emotional exhaustion and anger. This significant influence assimilates the role of discrete sentiments in an organizational setting, and it suggests that emotional management can resolve individual anger and exhaustion. Overall, our findings provided support for the conversation of resource theory and explained how emotional exhaustion mediates the relationship between workplace cyberbullying and interpersonal deviance. By endorsing affective event theory, we propose innovative perceptions of how sentiments function as an indispensable link between the harmful workplace and employee behavior; by doing so, this study exposes a black box of the consequences of adverse work environment on employee behaviors. We can hope that the proposed study assists as a platform for lingering inquiries of how managers can evade the hazard that deplorable workplace events, such as workplace cyberbullying, translate into interpersonal deviance that leads to an intensification of these praxes rather than engendering suitable resolutions to tenacity them.

## Data availability statement

The data analyzed in this study is available on upon request. Requests to access these datasets should be directed to MB, misbahbhatti76@yahoo.com.

## Ethics statement

Ethical review and approval was not required for the study on human participants in accordance with the local legislation and institutional requirements. Written informed consent from the patients/participants or patients/participants legal guardian/next of kin was not required to participate in this study in accordance with the national legislation and the institutional requirements.

## Author contributions

NS: original—writing and data collection. AH: supervision and review—editing. XS: data analysis and review. MB: review—editing and data collection. All authors contributed to the article and approved the submitted version.
